# The Gollop–Wolfgang Complex: A Case Report

**DOI:** 10.3390/pediatric17020047

**Published:** 2025-04-16

**Authors:** Jun-Bum Kim, Byung-Ryul Lee, Jong-Seok Park, Chang-Hwa Hong, Sai-Won Kwon, Woo-Jong Kim, Soon-Do Wang, Dong-Woo Lee, Kyeung-Min Nam, Ki-Jin Jung

**Affiliations:** Department of Orthopaedic Surgery, Soonchunhyang University Hospital Cheonan, 31, Suncheonhyang 6-gil, Dongnam-gu, Cheonan 31151, Republic of Korea; kjbos@schmc.ac.kr (J.-B.K.); 129027@sch.ac.kr (B.-R.L.); jsparksch@schmc.ac.kr (J.-S.P.); chhong@schmc.ac.kr (C.-H.H.); osos@schmc.ac.kr (S.-W.K.); 89489@schmc.ac.kr (W.-J.K.); 118539@schmc.ac.kr (S.-D.W.); 122923@schmc.ac.kr (D.-W.L.); 144256@schmc.ac.kr (K.-M.N.)

**Keywords:** bifid femur, whole genome sequencing, split-hand/foot malformation, hemimelia

## Abstract

**Background:** The Gollop–Wolfgang complex is a rare congenital limb deformity characterized by a bifid femur, tibial hemimelia, and ectrodactyly of the hand. First described in 1980, fewer than 200 cases have been reported globally, with an estimated incidence of 1:1,000,000 live births. **Case Presentation**: We report a 2-month-old female infant with classic features of the Gollop–Wolfgang complex, including a left bifid femur, complete absence of the left tibia, and contralateral tetradactyly. A clinical examination revealed significant limb length discrepancy, knee instability, equinovarus foot deformity, and skeletal abnormalities confirmed by imaging studies. Extensive investigations, including echocardiography and genetic testing, excluded systemic anomalies and identified non-pathogenic variants in the Collagen Type XI Alpha 2 (COL11A2) and EVC2 genes. A surgical resection of the bifid femur was performed. **Results**: This case highlights the importance of early diagnosis and a multidisciplinary approach in managing the Gollop–Wolfgang complex. While our case presented with typical features, subtle variations highlight the phenotypic spectrum of the condition. The combination of tibial hemimelia and bifid femur frequently necessitates knee disarticulation due to the absence of a viable tibial anlage, while limb salvage techniques remain challenging. A genetic evaluation identified variants of uncertain significance in the COL11A2 and EVC2 genes, indicating that the genetic basis of the condition is not fully understood. **Conclusions**: These findings emphasize the need for continued genetic research to clarify the etiology of the Gollop–Wolfgang complex and to improve treatment strategies, particularly in refining surgical approaches and exploring new therapeutic options.

## 1. Introduction

Congenital tibial hemimelia with ipsilateral bifurcation of the femur is a rare anomaly. This condition was first reported by Erlich in 1885. In 1980, Gollop et al. described a case involving two brothers with ectrodactyly of one hand, unilateral bifurcation of the femur, and the absence of both tibiae [1]. In 1984, Wolfgang et al. reported a single case of tibial hemimelia with ipsilateral femoral bifurcation and contralateral tibial diastasis [2]. In 1986, Lurie and Ilyina introduced the eponym Gollop–Wolfgang complex or bifurcated femora–hand ectrodactyly complex and concluded that the association between hand ectrodactyly and femoral bifurcation is not coincidental [3]. Additionally, many cases of Gollop–Wolfgang complex are associated with other congenital abnormalities, which often align with the VACTERL sequence—vertebral anomalies, anal atresia, cardiovascular defects (most commonly ventricular septal defect [VSD]), tracheoesophageal fistula, esophageal atresia, renal anomalies, and limb defects, particularly radial ray abnormalities [4].

According to the United States Office of Rare Diseases at the National Institutes of Health, only 200 cases of Gollop–Wolfgang complex have been reported to date. Gollop–Wolfgang complex reportedly has an incidence of 1:1,000,000 live births [5]. While the condition appears to affect both sexes, no consistent **sex predilection** has been documented in the literature to date. Most published cases report both male and female patients, suggesting a non-sex-linked pattern of occurrence [4,5,6]. Its etiology is postulated to involve errors in the complex genetic regulation of limb development; however, the exact cause remains unclear. While autosomal dominant inheritance patterns with reduced penetrance and variable expressivity are the most common patterns, other modes, including X-linked and autosomal recessive inheritance, have also been observed. Additionally, de novo mutations cannot be excluded, as evidenced by reported cases [7]. The genetic basis of the Gollop–Wolfgang complex is still not fully understood; however, recent advances in genomic technologies have shed more light on the molecular underpinnings of split-hand/foot malformations with long bone deficiency (SHFLD). Duplications of the *BHLHA9* gene on chromosome 17p13.3 have been strongly implicated in SHFLD syndromes, including the Gollop–Wolfgang complex. Odrzywolski et al. identified novel non-coding regulatory variants that affect *BHLHA9* expression during limb development, suggesting broader regulatory complexity [8]. Similarly, Tekin et al. highlighted genotype–phenotype correlations in a cohort with ectrodactyly and tibial anomalies, further supporting the role of *BHLHA9* duplications and epigenetic modifiers. These findings suggest that while *BHLHA9* remains the primary gene of interest, additional modifier genes and environmental interactions may contribute to the variable expressivity of the condition [9].

The present report describes the case of a patient with Gollop–Wolfgang syndrome who presented to our clinic with multiple musculoskeletal deformities.

## 2. Case Presentation

A 2-month-old female, born at 38 weeks via cesarean delivery because of breech presentation, was referred to our pediatric orthopedic clinic for the evaluation of a left femur and knee anomaly. The pregnancy was unremarkable, with regular ultrasound screenings. The infant was the first child of a 19-year-old Korean primigravida mother. The mother reported no history of drug, alcohol, or tobacco use, nor any diabetes mellitus or infections during pregnancy. No family history of consanguinity, birth defects, congenital abnormalities, or limb deficiencies were recorded.

**Figure 1:**
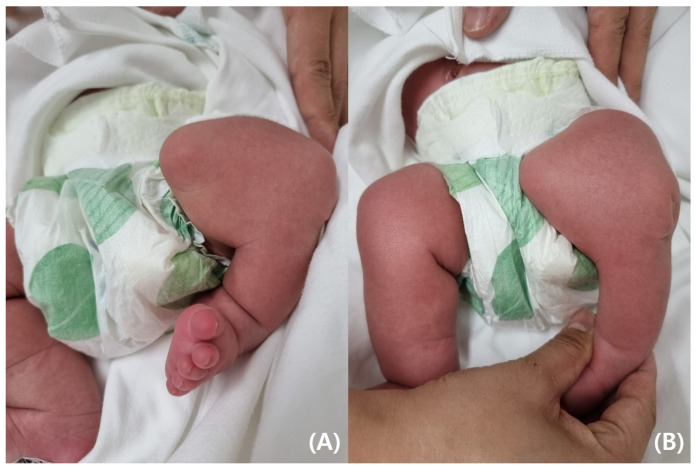
(**A**) Bony protuberance at the distal third of the anteromedial aspect of the left thigh. (**B**) Normally developed right lower limb.

**Figure 2:**
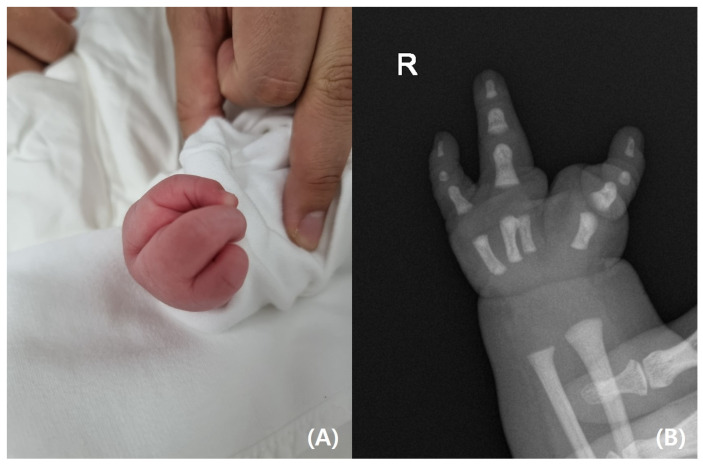
(**A**) Ectrodactyly of the right hand. (**B**) Radiograph of the right hand showing tetradactylous ectodactyly.

**Figure 3:**
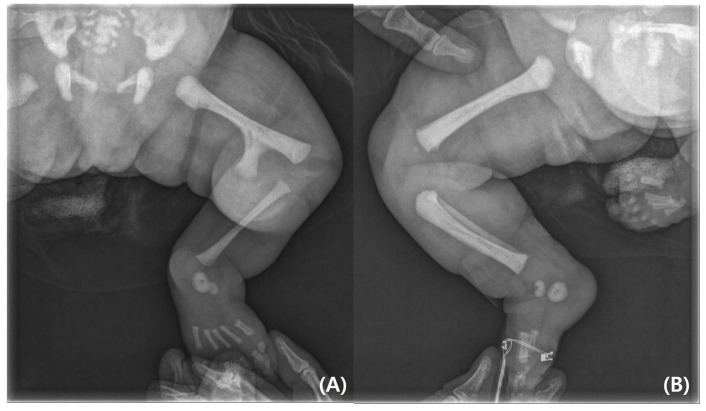
(**A**) Radiographs of lower limbs showing left distal femoral bifurcation, left tibial and patellar agenesis. (**B**) Normal femur, tibia, fibula, and patella on the right side.

On physical examination, the infant’s left leg was shorter than the right leg, and her left foot had equinovarus (Figure 1). A large bony prominence was observed on the medial aspect of the left thigh. The left knee joint was unstable in all directions. The left fibula was palpable just lateral to the knee and ankle joints. The tibia, including the medial malleolus, was not palpable. The feet were grossly deformed because of equinovarus. Her upper extremities revealed contralateral tetradactylous ectodactyly, and the left side showed normal development with equal length and normal digits (Figure 2). A radiography examination of the lower extremities revealed the absence of the left tibia, a deformed left foot, and duplication of the distal left femur. No epiphyseal ossification was observed at the distal end of the left femur (Figure 3). Ultrasonography of the same leg confirmed weak quadriceps and undetectable patellar signals and additionally revealed a complete absence of any tibial anlage in the lower leg. Neurologically, the patient was intact, and on examination of her lower back, the gluteal cleft was midline with no dermatological anomalies on her lower back.

A comprehensive skeletal survey was unremarkable for any associated anomalies. Subsequent investigations with echocardiography, abdominal ultrasonography, chest radiography, and laboratory biochemical tests were unremarkable. Pelvic and abdominal ultrasonography identified no renal or visceral abnormalities, and echocardiography conducted at the time of admission confirmed the absence of congenital cardiac defects. Tandem mass spectrometry revealed no evidence of congenital metabolic abnormalities.

A genetic analysis was conducted on the newborn using cytogenetic analysis, chromosomal microarray (CMA), and next-generation sequencing (NGS) for skeletal dysplasia. Peripheral venous blood samples were collected and transferred into ethylenediaminetetraacetic acid (EDTA) anticoagulant tubes. These samples were sent for genetic analysis at two central laboratories: SamKwang Medical Laboratories (Seoul, Republic of Korea) and GC Labs (Yongin-Si, Gyeonggi-do, Republic of Korea). Each institution’s qualified cytogeneticist or molecular pathologist generated an overall clinical laboratory interpretation for each sample, assessing chromosomal copy number variant (CNV) regions and classifying them as ‘pathogenic’, ‘likely pathogenic’, or ‘variant of unknown significance (VUS)’.

A conventional GTG-banding analysis was performed on 20 metaphase chromosomes obtained from the blood sample. The results indicated a normal chromosomal composition, with a reported fetal karyotype of 46, XX. Genomic DNA was analyzed using a chromosomal microarray analysis (CMA) with Human Genome Build 19 (Genome Reference Consortium GRCh37). The CytoScan Dx Assay was used to assess CNV coordinates, which were compared with the coordinates of actionable microarray findings. No structural anomalies associated with specific microdeletions or microduplications were detected.

Next-generation sequencing (NGS)-based targeted gene analysis was performed on 433 genes associated with skeletal dysplasia following the American College of Medical Genetics and Genomics (ACMG)’s guidelines [10]. Massively parallel sequencing was conducted using the Illumina NextSeq 500^®^ platform (Illumina Inc., San Diego, CA, USA). Variants with minor allele frequencies (MAFs) >1% in the 1000 Genomes Browser, Genome Aggregation Database (gnomAD), and Korean Reference Genome Database (KRGDB) were excluded. In silico analyses using SIFT, PolyPhen-2, and MutationTaster were performed to assess the potential impact of missense variants.

Panel testing identified variants in COL11A2 and EVC2, though their clinical significance remains uncertain. The COL11A2 c.4346C>G variant is extremely rare, absent from population databases (gnomAD and KRGDB). Computational predictions were conflicting: MutationTaster classified it as deleterious, while SIFT and PolyPhen-2 predicted it to be tolerated. Similarly, the EVC2 c.1631A>C variant had an MAF value of 0.0004% in the general population (gnomAD) and 0.0455% in the Korean population (KRGDB), with in silico models predicting a deleterious effect. Due to insufficient evidence linking these variants to disease, both were classified as variants of uncertain significance (VUS) (Table 1).

**Figure 4:**
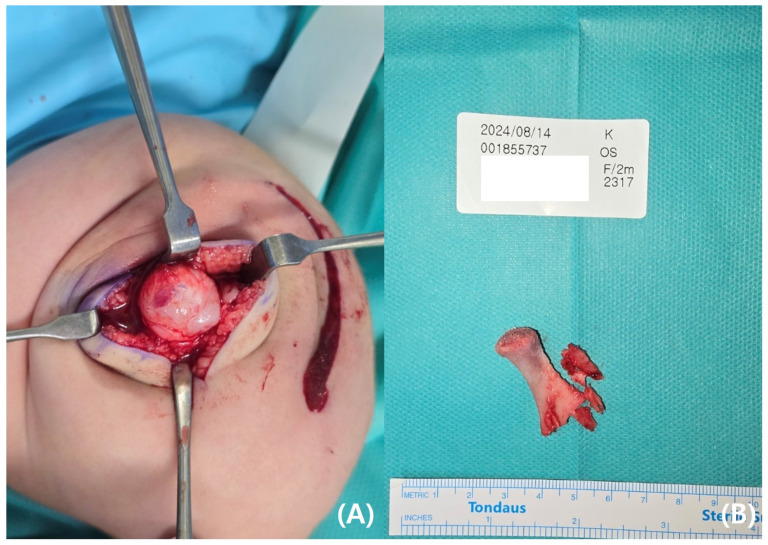
(**A**) Photographs of bony protuberance of left distal femur. (**B**) Excised bony protuberance.

**Figure 5:**
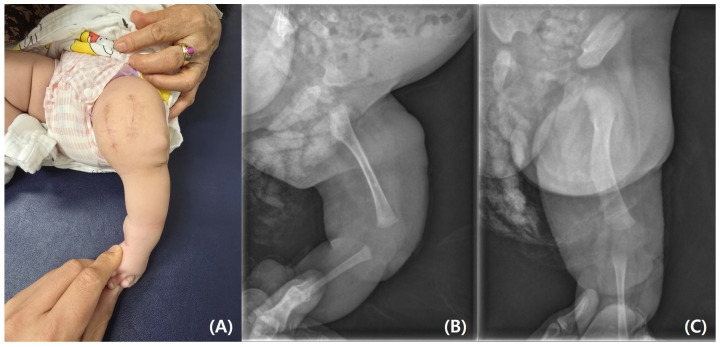
(**A**) Postoperative photograph. (**B**) Postoperative lateral and (**C**) anteroposterior radiographs.

The parents requested rapid resection of the bifid femur with aggressive treatment. At the age of 4 months, surgical excision of the left bifid femur was performed (Figure 4 and Figure 5). The bony protuberance of the distal femur was resected through an anteromedial incision. After resecting the bone, a tented, hard skin lump remained. While making another incision to resect the remaining skin, we found what appeared to be the cartilage cap, which was subsequently removed. The resected femur was sent for pathological examination, and the results showed no specific abnormalities. The patient’s course of recovery at the hospital was uncomplicated, and she was discharged on postoperative day 2. She was subsequently scheduled for follow-up care after discharge, including wound management and suture removal.

## 3. Discussion

The Gollop–Wolfgang complex is a rare congenital limb anomaly characterized by the association of distal bifid femur and tibial agenesis. Initially described by Gollop et al. in 1980, the condition was defined by unilateral femoral bifurcation, bilateral tibial absence, monodactyly of the feet, and ectrodactyly of one hand [1]. Subsequently, Wolfgang expanded the definition in 1984 to include right femoral bifurcation, tibia hemimelia, and contralateral tibial diastasis. However, Lurie and Ilyina clarified the definition in 1986 by excluding femoral bifurcation and hand ectrodactyly from the core features [2].

The Gollop–Wolfgang complex has been associated with various congenital abnormalities, often aligning with the VACTERL association. While an autosomal dominant inheritance pattern with complete penetrance is the most common mode of transmission, other patterns, such as X-linked and autosomal recessive, have been reported. Additionally, the possibility of de novo mutations cannot be excluded [4].

Tibial agenesis, a key component of the Gollop–Wolfgang complex, is a congenital deformity affecting ankles, knee joints, and adjacent musculotendinous units. The classification of tibial deficiency is essential for accurate diagnosis and surgical planning. Three major classification systems are widely used: those proposed by Kalamchi and Dawe, Jones, and Weber [11]. Among these, the Jones classification is the most commonly utilized and divides tibial agenesis into four types. It can be classified into types Ia and Ib based on radiographic findings, with type Ia representing complete tibial absence and type Ib indicating complete absence with a hypoplastic lower femoral epiphysis. Type 2 involves an ossified proximal tibia visible at birth, with a missing distal portion. Type 3, the least common, features an ossified distal tibia but absent proximal portion. Type 4 presents with a shortened tibia and distal tibiofibular diastasis [7]. The bifid femur, another characteristic feature, manifests as a more or less severe bowing of the upper leg, often accompanied by equinovarus deformity of the foot, knee anomalies, and patella absence. This condition frequently co-occurs with other congenital limb anomalies, such as ectrodactyly of the hands or feet, and systemic anomalies as seen in VACTERL syndrome [5].

The Gollop–Wolfgang complex, as exemplified in our case, encompasses the association of a bifid femur, tibial hemimelia, and hand ectrodactyly. According to the Jones classification, the present case corresponds to type Ia, characterized by complete absence of the tibia with no ossification center, along with a hypoplastic femur. Understanding the complex genetic and developmental factors underlying this condition is crucial for accurate diagnosis, genetic counseling, and appropriate surgical management.

To deepen the understanding of this complex condition, the following subsections address its potential causes, associated systemic abnormalities, diagnostic challenges during the prenatal period, and treatment strategies.

### 3.1. Etiology

Ectrodactyly, also known as split-hand or foot malformation (SHFM), is caused by abnormal developmental signaling during embryogenesis. SHFM with long-bone deficiency (SHFLD) is characterized by SHFM with additional long-bone deficiency, which typically affects the tibia. We described the case of the Gollop–Wolfgang complex, which is classified as SHFLD [12].

SHFLD is associated with the duplication of 17p13.3. SHFLD is reportedly inherited in an autosomal dominant or autosomal recessive manner, although there have been some cases of spontaneous development without a family history, as in the present case [13]. The minimal 17p13.3 critical region encompasses a single gene, BHLHA9. Klopocki et al. revealed a defect in tandem duplication and narrowed the region to a single gene, BHLHA9 [14]. The genomic duplication of BHLHA9 is recognized as the most common cause underlying the Gollop–Wolfgang complex [15]. However, in the present case, standard genetic testing—including cytogenetic analysis, chromosomal microarray, and next-generation sequencing (NGS) panel testing—did not identify a clear pathogenic variant. Although variants were found in COL11A2 and EVC2, their clinical significance is unclear. Further research is needed to determine if these variants play a role in SHFLD.

While no definitive risk factors have been identified, several possible etiologies have been proposed in the medical literature. Many studies suggest a primary genetic defect, while others have highlighted the potential role of antiepileptic drugs, particularly carbamazepine and valproic acid, used during pregnancy as contributing risk factors. Comprehensive elucidation of the etiology requires a thorough analysis encompassing all reported cases, with a detailed comparison of genetic findings and familial histories to better understand the underlying mechanisms of this complex condition [16].

### 3.2. Extraosseus Manifestation

Although the skeletal limb manifestations of the Gollop–Wolfgang complex are well described, it is crucial to identify the extraosseous malformations observed in this syndrome [1,2]. Among the extraosseous manifestations, cardiovascular disease (17%), including atrial and ventral septal defects, is the most common. Gastrointestinal abnormalities (12%), such as the tracheoesophageal fistula, as well as pulmonary, renal, and genitourinary system issues, have all been described in the literature [17]. Raas-Rothschild et al. and van de Kamp et al. observed cardiac anomalies in infants diagnosed with Gollop–Wolfgang complex and explored its potential connection to VACTERL syndrome [18]. Additionally, Evans and Chudley reviewed eight cases of individuals with Gollop–Wolfgang complex accompanied by caudal midline defects, including their own reported case. Therefore, the potential for such associated conditions should not be overlooked in the Gollop–Wolfgang complex [5].

### 3.3. Prenatal Evaluation

Early prenatal diagnosis allows for detailed multidisciplinary counseling to the parents and anticipation of different clinical scenarios that may occur at birth, along with specific treatment options [19]. Skeletal deformities are usually detectable between 16 and 22 weeks of gestation, with a short femur often being the first clue on ultrasonography. While prenatal diagnosis is technically feasible during the anomaly scan, it can be challenging because of intrinsic limitations or technical issues such as fetal position, reduced amniotic fluid, or maternal obesity [20]. Our patient underwent regular prenatal check-ups, including ultrasound screenings; however, malformations were not detected in advance. This could potentially be attributed to poor fetal positioning or technical limitations during the scan, such as suboptimal angles, limb overlap, or insufficient visualization of long bones. However, considering the severity and size of the deformities, the possibility of a missed diagnosis due to operator error or interpretation limitations cannot be excluded. Therefore, it may be more accurate to consider both technical challenges and diagnostic oversight as contributing factors to prenatal non-detection.

A retrospective review of sonographic studies was performed at two fetal care centers in the United States and China from 2002 to 2012. Fetal abnormalities consistent with SHFM were identified in ten pregnant women using ultrasonography. The gestational age of the fetuses at the time of diagnosis ranged from 15 to 29 weeks. Seven out of ten pregnancies were electively terminated because of malformations, while three women delivered at term. Considering the high termination rate, the actual occurrence rate of malformations is thought to be higher than reported [21].

### 3.4. Treatment

Managing the Gollop–Wolfgang complex requires prior knowledge of any tibial anlage because it carries far-reaching therapeutic consequences [22]. Therefore, ultrasonography or magnetic resonance imaging of the lower leg is recommended. If a cartilaginous tibial anlage is identified, reconstruction of the lower leg should be considered, assuming that this cartilaginous remnant will ossify later, enabling good active extension. In case of a total absence of the tibia without an extensor mechanism, as observed in our patient, exarticulation through the knee followed by fitting with a modern prosthesis is the best course of action [23]. Alternative approaches, such as femorofibular fusion and fibular tibialization, are available but have inconsistent success rates [23,24].

Advances in prosthetics and reconstruction, especially with a patella, offer hope for better function, potentially reducing the need for amputation. While limb salvage may be an option for those opposing amputation for cultural or religious reasons, it has unpredictable outcomes, whereas in the absence of a proximal tibial anlage, knee disarticulation remains the best treatment option and should be performed as soon as possible [17,22,25]. Early amputation has been associated with increased activity levels, reduced pain, and higher patient satisfaction [26].

Fibular transfer with ankle fusion has been accepted as a limb salvage procedure; however, previous reports have described relatively poor results for fibular transfer because of marked knee instability and the progressive development of knee flexion contracture owing to insufficient quadricep strength. Loder and Herring reported that 53 of 55 patients with complete tibial hemimelia who underwent fibular transfer had poor outcomes [23,25,27]. Furthermore, Schoenecker et al. reported secondary amputations after fibular transfer in >50% of their cases [28]. Consequently, primary knee disarticulation was preferred.

Looking toward the future, further research into gene therapy and genetic editing techniques holds considerable promise for treating this condition by correcting defects at the cellular level.

## 4. Conclusions

There were several findings in both the clinical and radiographic presentations between our case and previously documented cases of the Gollop–Wolfgang complex. The characteristic presence of tibial hemimelia and bifid femur often necessitates knee disarticulation. This highlights the importance of continued research into more effective treatment options. Additionally, the identification of variants of uncertain significance in COL11A2 and EVC2 through genetic evaluation further emphasizes the critical role of ongoing genetic investigation in understanding this complex condition.

## Figures and Tables

**Table 1:** Results of hereditary skeletal dysplasia NGS panel.

GENE	DNA CHANGE	PREDICTED AA CHANGE	ZYGOSITY	OMIM DISEASE	INHERIT	CLASS
COL11A2	c.4346C>G	p.Pro1449Arg	Het	D13/53, OSMED	AD, AR	VUS
EVC2	c.1631A>C	p.Gin544Pro	Het	EVC, WAD	AR	VUS

Reference sequence: NM_080680.3(COL11A2): NM_147127.5(EVC2). OMM disease: D13/53, deafness 13/53; OSMED, Otospondylomegaepiphyseal dysplasia; EVC, Elis-van Creveld syndrome; WAD, Weyers acrofacial dysostosis. Abbreviations: Het, heterozygous; AD, autosomal dominant; AR, autosomal recessive; VUS, variant of uncertain significance.

## Data Availability

The datasets used and/or analyzed during the current study are available from the corresponding author upon reasonable request.
